# Antinociceptive Activity of *Macaranga denticulata* Muell. Arg. (Family: Euphorbiaceae): *In Vivo* and *In Silico* Studies

**DOI:** 10.3390/medicines4040088

**Published:** 2017-12-01

**Authors:** Abul Hasanat, Tanvir Ahmad Chowdhury, Mohammad Shah Hafez Kabir, Mohammed Sohel Chowdhury, Md. Nazim Uddin Chy, Jackie Barua, Nishan Chakrabarty, Arkajyoti Paul

**Affiliations:** 1 Department of Pharmacy, International Islamic University Chittagong, Chittagong 4318, Bangladesh; pharmahasanat@gmail.com (A.H.); tanvirpharma31@gmail.com (T.A.C.); mohammadshahhafezkabir@yahoo.com (M.S.H.K.); 1349sohelas@gmail.com (M.S.C.); nazim107282@gmail.com (M.N.U.C.); nishaniiuc@gmail.com (N.C.); 2 GUSTO A Research Group, Chittagong 4000, Bangladesh; jackiebarua1884@gmail.com; 3 Comilla Medical College, Faculty of Medicine, University of Chittagong, Chittagong 4331, Bangladesh; 4 Department of Microbiology, Jagannath University, Dhaka 1100, Bangladesh

**Keywords:** *Macaranga denticulata*, COX-1, molecular docking, antinociceptive activity

## Abstract

**Background:** The present study was conducted to investigate the antinociceptive activity of methanol extract of *Macaranga denticulata* (Met.MD) in an animal model, followed by molecular docking analysis. **Methods:** Antinociceptive activity was determined by acetic acid-induced writhing and formalin-induced licking test in mice. Then, molecular docking study was performed to identify compounds having maximum activity against the COX-1 enzyme using Schrödinger Maestro (version 10.1) to determine docking fitness. **Results:** A preliminary phytochemical analysis of Met.MD revealed that it contained alkaloids, carbohydrates, phenols, flavonoids, tannins, and terpenoids. Met.MD exhibited a dose-dependent and statistically significant antinociceptive activity in the acetic acid and formalin test at the doses of 200 and 400 mg/kg. In addition, our docking study showed that macarangin had the best fitness score of −5.81 with COX-1 enzyme among six major compounds of *M. denticulata*. **Conclusions:** Results of the present study confirmed the potential antinociceptive activity of *M. denticulata* leaf extract in both *in vivo* and *in silico* models.

## 1. Introduction

*Macaranga denticulata* Muell. Arg. (Family: Euphorbiaceae), locally known as Burna or Burakochi, is a low evergreen tree grown throughout Asia, mainly in Bangladesh and Northern Thailand. It is widely distributed in the Chittagong hill tracts area of Bangladesh. Different parts of the plant are usedfor various ailments in the traditional medicine of the country. The leaf is considered the most common serving part of this plant for its ability to alleviate constipation, mucous stool, and colic [1]. Furthermore, fresh or dried leaves of some *Macaranga* species are used to treat swellings, cuts, sores, boils, and bruises in folk medicine. The preliminary phytochemical study revealed that the genus of *Macaranga*is enriched with flavonoids (isoprenylated, geranylated, and farnesylated flavonoids) and stilbenes. Other than flavonoids, it also contains terpenes, tannins, and coumarins [2]. Researchers have isolated several phytomolecules from *M. denticulata* such as 3-acetylaleuritolic acid, oleanolic acid, macarangin, scopoletin, β-sitosterol, and stigmasterol [3,4,5]. Pharmacological properties, such as thrombolytic, cytotoxic, anti-arthritic, antibacterial, and in silico PASS prediction activities, have been reported [2,6].

However, there has not been any report demonstrating the antinociceptive activity of the leaves of *M. denticulata*. Keeping this in view, the objective of the present study was to investigate this in both *in vivo* and *in silico* models. 

## 2. Methods and Materials

### 2.1. Plant Sample Collection and Identification

Fresh leaves of *Macaranga denticulata* were collected from the Chittagong city area in front of Chittagong Medical college hostel gate of Chittagong, Bangladesh, in September 2014. Taxonomic identification was made by Dr. Shaikh Bokhtear Uddin, a botanist at the Department of Botany, University of Chittagong (CU), Chittagong 4331, Bangladesh, against the voucher specimen with the reference number of 16018. The sample has been deposited in the Department of Pharmacy, International Islamic University Chittagong (IIUC) for future reference.

### 2.2. Extraction Procedure

The collected fresh leaves were washed, cut into small parts, dried in the shade, and finally ground into coarse powder. The powdered plant material (about 450 g) was taken in a clean, flat-bottomed glass container and soaked in 900 mL of methanol. The glass container with its contents was clogged and retained for a period of 14 days with frequent stirring and shaking. The entire mixture was filtered by a piece of clean and white sterilized cotton materials followed by Whatman No.1 filter paper. Finally, the filtrate solution was evaporated to yield the methanol extract of *M. denticulata* (Met.MD = 13 g), which was then kept in a refrigerator at 4 °C until further use. The crude methanol extract showed a yield of 2.88%.

### 2.3. Drugs and Chemicals

Diclofenac sodium was obtained from Square Pharmaceuticals Ltd., Pabna, Bangladesh. Formalin and acetic acid were purchased from Merck (Darmstadt, Germany). Tween 80 was from(BDH Chemicals, Hunter Boulevard, Magna Park, Lutterworth, Leicestershire, UK), and the rest of the chemicals used were of analytical grade and from BDH, Fluka Chemie GmbH (Buchs, Switzerland) and Merck (Darmstadt, Germany).

### 2.4. Swiss Albino Mice 

Swiss albino mice (both sex) weighing approximately 25–35 g were used for this experiment. The mice were collected from Jahangir Nagar University, Savar (Manikganj Highway), Dhaka 1342, Bangladesh. The test animals were maintained in standard laboratory conditions (room temperature 25 ± 2 °C; 55–60% relative humidity; 12 h light/dark cycle) and provided with standard laboratory food and distilled water ad libitum. All experiments were conducted in noiseless conditions, and the animals were acclimatized to laboratory conditions for 10 days before experimentation. The current study protocol was reviewed and approved by the “Planning & Development Committee” of the Department of Pharmacy, International Islamic University Chittagong, Bangladesh under the name Pharm-P&D-61/08’14-125.

### 2.5. Phytochemical Screening

Qualitative phytochemical analysis of the extracts was carried out to determine the presence of alkaloids, carbohydrates, phenolic compounds, flavonoids, tannins, and terpenoids, respectively, as described previously [7].

### 2.6. Acute Toxicity Study

Acute toxicity study of Met.MD was observed in four experimental groups. Each group contained six animals (*n* = 6). The experimental groups received Met.MD at doses of 500, 800, 1000, to 2000 mg/kg body weight orally. Each group of Swiss albino mice were placed in separate cages and allowed free access to water ad libitum and food. The animals were observed for the next 72 h to find any mortality, adverse reactions such as skin rashes, itching, swelling, and behavioral changes [8].

### 2.7. In Vivo Antinociceptive Activity

#### 2.7.1. Acetic Acid-Induced Writhing Test

The acetic acid-induced writhing test was performed according to a previously reported method [9] with some modifications for the evaluation of the analgesic potential of Met.MD. In this study, Group I was administered 1% Tween-80 in distilled water which served as control; Group II was administered diclofenac sodium (10 mg/kg);Groups III and IV were administered 200 and 400 mg/kg (body weight, p.o) of Met.MD, respectively. After 30 min, 0.6% acetic acid (10 mL/kg) was injected intraperitoneally (i.p) into the test model (mice). The number of writhings (contraction of the abdomen, twisting of the mice trunk, elongation, an extension of body and limbs) was counted for 5–30 min after the administration of acetic acid. Antinociceptive activity was expressed as the percent of writhing inhibition (%) and calculated using the following formula:

% inhibition = [(C − T)/C] × 100

where C is the mean number of writhing of the control group, and T is the mean number of writhing of the test sample.

#### 2.7.2. Formalin-Induced Licking Test

The formalin-induced licking test was performed according to a previously reported method with some modifications [10]. After 30 min, 20 µL of 2.5% (*v*/*v* in distilled water) formalin was injected subcutaneously into the plantar surface of the right hind paw of the mice. In this study, Group I was administered 1% Tween-80 in distilled water, which served as control; Group II was administered diclofenac sodium (10 mg/kg);Groups III and IV were administered 200 and 400 mg/kg (body weight, p.o) of Met.MD, respectively. The formalin-induced licking of the paw was considered as indicative of nociceptive behavior. The total durationof behavioral responses to nociception, including licking and biting of the injected paw, was recorded for up to 30 min, where the first 5 min was considered as the early phase (neurogenic phase) and the second period of 15–30 min as the late phase (inflammatory phase) of the nociceptive response. Antinociceptive activity was expressed as the percentage inhibition of licking time and calculated using the following formula:

% inhibition = [(C − T)/C] × 100

where C is the licking time of the control group in seconds, and T is the licking time of the test sample in seconds.

### 2.8. In Silico Molecular Docking Study

For the molecular docking study, Glide of Schrödinger Maestro (version 10.1, Schrödinger, LLC New York, NY, USA) was used to predict the potent active compound *M. denticulata* against the active site of COX-1 enzyme, compounds of which werecollected from the literature review.

#### 2.8.1. Ligand and Protein Preparation 

The chemical structures of six major compounds isolated from *Macaranga denticulata*, namely, 3-acetyl aleuritolic acid (PubChem CID: 161616), β-Sitosterol (PubChem CID: 222284), macarangin (PubChem CID: 10047854), oleanolic acid (PubChem CID: 10494), scopoletin (PubChem CID: 5280460), and stigmasterol (PubChem CID: 5280794) were obtained from the PubChem Project database and were structurally plotted in 3 dimensions (3D) using Ligprep 2.5 in Schrödinger Suite, 2013, and their ionization states were generated at pH (7.0 ± 2.0) using Epik 2.2 in Schrödinger Suite. In case of the protein preparation, the 3D structure of the COX-1 enzyme was obtained from the Protein Data Bank (PDB, #2OYE) [11]. Afterward, the structure was prepared and refined using the protein preparation wizard (Schrödinger Maestro (version 10.1)), where charges and bond orders were assigned, hydrogens were added to the heavy atoms, selenomethionines were converted to methionine, and all water portions were removed. On the one hand, certain hydroxyl and thiol groups were reoriented, and on the other, amide groups of glutamine, asparagines, the imidazole ring of histidines, the protonation states of histidines, and glutamic acidand aspartic acids were optimized at neutral pH. Using force field OPLS_2005, minimization was carried out with the maximum heavy atom RMSD set to 0.30 Å.

#### 2.8.2. Receptor Grid Generation

In Glide, grids were generated keeping the default parameters of the van der Waals scaling factor 1.00 and charge cut-off 0.25 subjected to theOPLS_2005 force field. A cubic box of specific dimensions centered around the centroid of the active site residues (ligand activation site) was generated for the receptor. The bounding box was set to 16 Å × 16 Å × 16 Å for docking experiments. It is necessary to identify the active binding site in the target protein.

#### 2.8.3. Glide Standard Precision Ligand Docking

SP flexible ligand docking was carried out in Glide of Schrödinger Maestro (version 10.1) [12,13] within which penalties were applied to non-cis/trans amide bonds. Glide standard precision (SP) docking was performed with these molecules, and hits above 4 kcal/mole based on a docking score were are-dockedCOX-1 enzyme in XP mode, keeping all docking parameters as default. No bonding constraints were given during docking calculations. Using a Monte Carlo random search algorithm, ligand poses were generated for each input molecule, and the binding affinity of these molecules to the COX-1 enzyme was predicted with respect tothe Glide docking score. 

Potential energies of the docked molecules were also predicted with an empirical E model scoring function. Post-dockingminimization was performed with the OPLS_2005 force field, and one pose per ligand was saved. Strain energies of bound and free forms of ligands were calculated, and hits with more than 4 kcal/mole energy difference between the two forms received a penalty equal to the quarter of their strain energy difference, which wasadded to the docking score.

### 2.9. Statistical Analysis

Values are presented as mean ± SEM. Data analysis among the groups was compared using one-way ANOVA followed by Dunnett’s test, where *p*-value less than 0.05 was considered to be statistically significant.

## 3. Results

### 3.1. Phytochemical Screening

Phytochemical screening of Met.MD revealed the presence of alkaloids, carbohydrates, phenolic compounds, flavonoids, tannins, and terpenoids.

### 3.2. Acute Toxicity Study

Oral administration of even the highest Met.MD dose, that is, 2000 mg/kg, did not produce any toxic effects in mice. Moreover, no mortality and behavioral change were recorded at the specified doses during the 72 h observation period. Thus, the extract was found to be safe at the given doses.

### 3.3. In Vivo Antinociceptive Activity: Acetic Acid Test

Met.MD, compared to the control, showed 43.26% and 57.30% greater inhibition of writhing at the doses of 200 and 400 mg/kg body weight, respectively, whereas diclofenac sodium, compared to the control, showed 69.66% greater inhibition at the dose of 10 mg/kg, and these results were statistically significant (Table 1).

### 3.4. In Vivo Antinociceptive Activity: Formalin Test

The effect of methanol extract of *M. denticulata* leaves at 200 and 400 mg/kg doses in the formalin test are shown in Table 2. At both doses, there was a dose-dependent decrease of paw licking time in the early phase, but a dose of 400 mg/kg significantly (*p* < 0.01) reduced latency to discomfort in the late phase compared to the late phase of the control. In contrast, the reference drug Diclofenac-Na (10 mg/kg) significantly reduced the licking activity against both phases of formalin-induced nociception.

### 3.5. In Silico Study: Molecular Docking Study for Antinociceptive Activity

**Figure 1:**
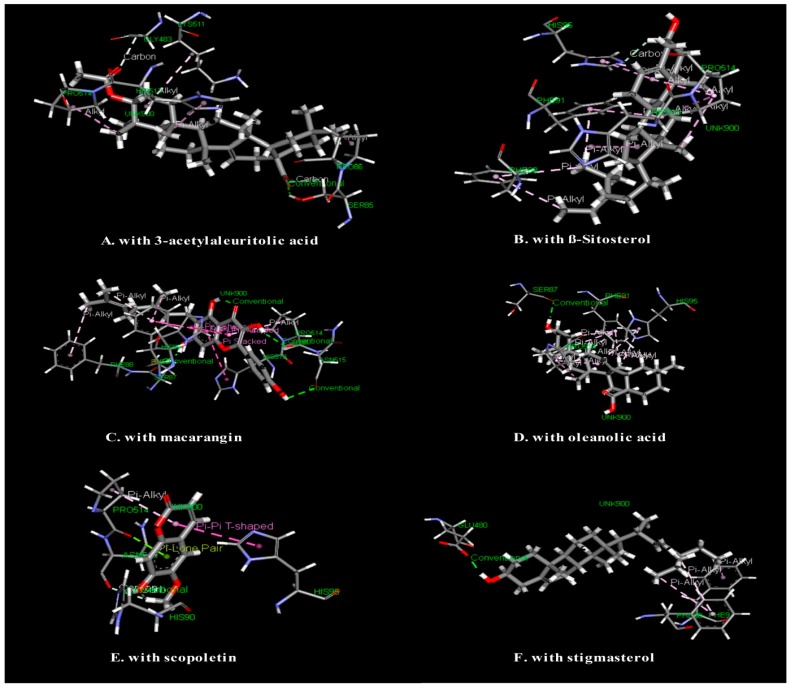
Visualization of the molecular docking interaction of isolated compounds of *M. denticulata* with COX-1 enzyme.

In this study, six major compounds isolated from *M. denticulata* were selected for molecular docking and the results of all ligands are described in Table 3. The molecular docking study showed that macarangin has the best glide fitness score against the COX-1 enzyme, which is −5.816. Interactions between ligands and COX-1 enzyme were presented in Figure 1.

## 4. Discussion

Medicinal plants are one of the most important sources of analgesics which act on centrally or peripherally since some clinically useful analgesic drugs have been discovered from plants, such as aspirin and morphine [14]. From this view, extensive studies are required to explore new therapeutic analgesic agents from natural sources. In the present study, we investigated the antinociceptive effect of the methanol extract of *M. denticulata* (Met.MD) leaves in different pain models followed by *in silico* molecular docking study.

For the experimental evaluation of antinociceptive activity, we began our investigation with an acetic acid-induced writhing inhibition test. This test has been used for the evaluation of peripheral antinociceptive activity [15]. The abdominal constrictions produced after the administration of acetic acid areconsidered as a visceral inflammatory pain as they impel capillary permeability and release endogenous substances (such as prostaglandins, histamine, cytokines, serotonin, cyclooxygenase, and COX) that excite pain nerve endings [16]. Here, Met.MD significantly inhibited mice abdominal writhing in a dose-dependent fashion compared to the control group. This suggests that the antinociceptive activity of Met.MD is strong and may be involved in the inhibition of the release of inflammatory mediators or in the direct blockage of receptors. 

Secondly, we performed a formalin-induced licking test since this test is a widely accepted method for distinguishing between central and peripheral antinociceptive activities. The administration of formalin into the right hind paw on mice produces two distinct phases, namely, the early phase (neurogenic phase) and the late phase (inflammatory phase) [17]. The early phase results from the direct stimulation of nociceptors, whereas the late phase involves a period of sensitization during which inflammatory phenomena occur [18]. The results of the present study show that Met.MD decreased the licking time in the neurogenic and inflammatory phases of formalin induction. A decrease in licking time in both phases indicates that it possesses both central and peripheral antinociceptive effects.

A preliminary phytochemical screening of Met.MD qualitatively identified the presence of alkaloids, carbohydrates, phenols, flavonoids, tannins, and terpenoids. Flavonoids have been reported to suppress intracellular Ca^2+^ level elevation and the release of pro-inflammatory mediators (such as histamine, serotonin, and TNFα) [16]. Flavonoids, tannins, and terpenoids have also been reported for antinociceptive activity [19]. It could, therefore, be suggested that the presence of these phytochemical constituents in the extract is responsible for the observed pharmacological effects. In addition, the preliminary acute toxicity test showed no occurrence of death or abnormal behavior at doses of 500–2000 mg/kg, suggesting that Met.MD possesses a low toxicity profile. 

To accelerate new drug discovery and development from natural products, a computer-aided drug design plays a vital role and has become an indispensable tool in the pharmaceutical industry [20]. From this perspective, we performed molecular docking (ligand–protein docking) since it is a key tool in structural molecular biology and computer-assisted drug design. The aim of ligand–protein docking is to predict the predominant binding mode(s) of a ligand with a protein of known three-dimensional structure based on the scoring function [21]. In this study, six major compounds of *M. denticulata* were docked into the active site of the COX-1 enzyme by means of Glide docking. Our current study showed that macarangin and scopoletin have the best fitness score of −5.81 and −5.38 with the COX-1 enzyme, respectively, which also suggests that they have a greater interaction with the COX-1 enzyme than the other compounds. Further studies are necessary to validate the pharmacological effects of these isolated compounds. 

## 5. Conclusions

The results of the present study confirmed the potential antinociceptive activity of *M. denticulata* leaf extract in an animal model, and its phytoconstituents could be a potential source of analgesic agents. Therefore, further studies are necessary to experimentally validate the pharmacological effects of these isolated compounds.

## Figures and Tables

**Table 1:** Antinociceptive effect of methanol extract of *M. denticulata* leaves in acetic acid-induced abdominal writhing test in mice.

Treatment	Writhing	% Inhibition
Control	59.33 ± 1.84	-
Diclofenac-Na (10 mg/kg)	18.00 ± 0.82 **	69.66
Met.MD (200 mg/kg)	33.67 ± 1.18 *	43.26
Met.MD (400 mg/kg)	25.33 ± 0.82 **	57.30

Met.MD = Methanol extract of *M. denticulata*; Values are presented as mean ± SEM (*n* = 6). * *p* < 0.05 and ** *p* < 0.01 compared with the control group (Dunnett’s test).

**Table 2:** Antinociceptive effect of methanol extract of *M. denticulata* leaves in formalin-induced licking test in mice.

Treatment	Early Phase (s)	% Inhibition of Early Phase	Late Phase (s)	% Inhibition of Late Phase
Control	57.31 ± 1.06	-	41.74 ± 1.46	-
Diclofenac-Na (10 mg/kg)	14.95 ± 0.60 **	73.91	12.26 ± 0.52 **	70.62
Met.MD (200 mg/kg)	30.47 ± 1.82 **	46.83	25.58 ± 1.56 *	38.72
Met.MD (400 mg/kg)	21.70 ± 1.16 **	62.14	21.36 ± 0.92 **	48.83

Met.MD = Methanol extract of *M. denticulata*; Values are presented as mean ± SEM (*n* = 6). * *p* < 0.05 and ** *p* < 0.01 compared with the control group (Dunnett’s test).

**Table 3:** Molecular docking score of the phytoconstitiuents isolated from *M. denticulata* on COX-1 enzyme for antinociceptive activity.

Compound Name	Molecular Docking Score	Glide E Model	Glide G Score
3-acetylaleuritolic acid	−2.226	−32.351	−2.232
β-Sitosterol	−3.024	−21.251	−3.024
macarangin	**−5.816**	−51.977	−5.816
oleanolic acid	−0.023	−24.329	−2.700
scopoletin	−5.381	−31.695	−5.386
stigmasterol	−2.397	−19.423	−2.397

Bold text indicates the highest docking score.
